# The Fructoborates: Part of a Family of Naturally Occurring Sugar–Borate Complexes—Biochemistry, Physiology, and Impact on Human Health: a Review

**DOI:** 10.1007/s12011-018-1550-4

**Published:** 2018-10-20

**Authors:** John M. Hunter, Boris V. Nemzer, Nagendra Rangavajla, Andrei Biţă, Otilia Constantina Rogoveanu, Johny Neamţu, Ion Romulus Scorei, Ludovic Everard Bejenaru, Gabriela Rău, Cornelia Bejenaru, George Dan Mogoşanu

**Affiliations:** 1 VDF FutureCeuticals, 2692 North State Route 1–17, Momence, IL 60954 USA; 2 Department of Pharmacognosy & Phytotherapy, Faculty of Pharmacy, University of Medicine and Pharmacy of Craiova, 2 Petru Rareş Street, 200349 Craiova, Romania; 3 Department of Physical Medicine and Rehabilitation, Faculty of Medicine, University of Medicine and Pharmacy of Craiova, 2 Petru Rareş Street, 200349 Craiova, Romania; 4 Department of Physics, Faculty of Pharmacy, University of Medicine and Pharmacy of Craiova, 2 Petru Rareş Street, 200349 Craiova, Romania; 5 BioBoron Research Institute, S.C. Natural Research S.R.L., 31B Dunării Street, 207465 Podari Commune, Dolj County, Romania; 6 Department of Organic Chemistry, Faculty of Pharmacy, University of Medicine and Pharmacy of Craiova, 2 Petru Rareş Street, 200349 Craiova, Romania; 7 Department of Vegetal & Animal Biology, Faculty of Pharmacy, University of Medicine and Pharmacy of Craiova, 2 Petru Rareş Street, 200349 Craiova, Romania

**Keywords:** Sugar–borate esters, Calcium fructoborate, Dietary supplements, Knee discomfort, Mechanisms of action

## Abstract

Sugar–borates (SBs) are mono- or di-sugar–borate esters (SBEs) comprised of one or two monosaccharide molecules linked to a boron (B) atom. SBEs occur naturally in commonly consumed herbs, vegetables, fruits, seeds, and nuts and, other than greatly varying levels of B found in local drinking water, are the primary natural dietary sources of B-containing molecules in humans. To date, the most studied SBE is calcium fructoborate (CaFB). CaFB represents an important example of how organic B-containing molecules are significantly distinct from their inorganic counterparts. During these past two decades, CaFB has been researched for its physical and biochemical characteristics, safety, and clinical outcomes. Results of these researches are presented and discussed herein. CaFB has been characterized using Fourier-transform infrared (FTIR) spectroscopy, thermogravimetric analysis (TGA), high-performance thin-layer chromatography (HPTLC), nuclear magnetic resonance (NMR), liquid chromatography–multistage accurate mass spectrometry (LC-MSn), X-ray diffraction (XRD), Raman spectroscopy, and inductively coupled plasma (ICP) in non-biological and biological specimens. Potential health benefits of CaFB have been clinically investigated in pilot and efficacy studies demonstrating (i) significant reductions in knee discomfort and improved flexibility within 7, 14, and 90 days and (ii) significant effect on blood levels of inflammatory, cardiovascular, and other biomarkers. These studies support the use of CaFB as a dietary supplement for the management of joint discomfort. CaFB is presented here in order to illustrate how physiological benefits are imparted by distinct organic boron-containing molecules rather than solely by the element B itself. Considering recent National Health and Nutrition Examination Survey (NHANES) data reporting increases in age-related joint pain and an increasing elderly demographic, SBEs offer potential for safe, natural, and effective management of joint discomfort and improved mobility in human and animal health applications. Several of these studies may also open new opportunities for use of SBEs for health benefits beyond joint health.

## Introduction

Of all the known elements, boron (B), after carbon, has perhaps the most interesting speciation, due to the fact that it can be found so frequently throughout nature. An element’s “speciation” is defined as the dispersion of that element amidst distinguished chemical species in a system [1]. B’s organic speciation in the three kingdoms of life (*Archaea*, *Bacteria*, and *Eukarya*) derives from the additional biochemical development of boron’s inorganic species [2–4]. Organic B biochemical species have formed the basis of B’s proven essentiality in plants [5, 6]. More recently, B has been suggested to be essential in animals [7, 8]. Such suggestion has not yet been conclusively proven, although many hypotheses have been postulated [4, 9]. B has the ability to complex with a variety of *cis*-diol biomolecules; however, not all possible B complexes occur in nature [10, 11]. Recent scientific data strongly suggests that only plants have the capacity to metabolize boric acid (BA)/borate (inorganic B speciation) and to transform it into sugar–borate esters (organic B speciation). Currently, it appears that humans and other animals do not have this capacity [4, 12]. Generally, as boric acid/borate enters the human/animal cell, it blocks other biomolecules that contain *cis*-diols, for example, nicotinamide adenine dinucleotide (NAD), *S*-adenosyl methionine (SAM), flavin adenine dinucleotide (FAD), and ribonuclease (RNase) [4, 13]. Sugar–borate complexes that occur naturally in plants are perhaps the most recognized biogenic *cis*-diol organic B species [4, 14–20]. Various surveys [21–24] have demonstrated that sugar–borates are crucial in the development of plants (for example, the normal cell wall growth, membrane functions, and the biosynthesis of lignin), but more recently, sugar–borates have been demonstrated to be potentially important modulators of human health [17, 18, 25, 26]. The identification of health benefits related to sugar–borates and the subsequent clinical validation of their significance has become an important new extension of boron science. Thus far, out of all sugar–borate esters, only fructoborate (calcium fructoborate—CaFB) has been clinically proven to have measurable activity in humans [4, 17, 19, 27–30].

A main focus of this review is to distinguish and differentiate the importance of the organic B species found in nature. To date, many B scholars have exhibited a tendency to describe various B species as “forms of boron.” We respectfully suggest here that such terminology is perhaps an unfortunate misnomer due to the inherent implication that any activity of a given B-containing molecule is imparted solely by B rather than by the unique synergistic chemistry of the molecular components. B-containing molecules are indeed vital to our existence, but the organic B species are arguably more influential in animal biosystems than inorganic B species or free B itself. Consequently, in support of this argument, a particular emphasis will be placed herein on positioning sugar–borate esters versus inorganic B species (also found in nature), in terms of their relative safety, dietary importance, and potential for health benefits. Concurrently, this paper will audit previous and ongoing research related to the bioavailability and clinical effectiveness of sugar–borate esters (SBEs), especially the CaFB complex. In view of exceptionally encouraging information thus far, the authors envision numerous significant opportunities for future research and applications in health and medicine for the sugar–borates.

## Natural Occurrence and Intake

The uptake, transport, and ultimate function of B in plants clearly illustrate the importance of the plant formation of B complexes containing sugars [7, 31]. The SBEs are a class of boron-carbohydrate phytochemicals that bear a moderately low amount of B [11, 16, 31, 32]. SBEs are naturally occurring and have been discovered in herbs, vegetables, fruits, seeds, and nuts common to the human diet [19, 20, 33]. The main physiologically stable “phyto” SBEs are pectic polysaccharide rhamnogalacturonan II (RG-II) [34], glucose and fructose borate esters, *bis*-sucrose borate esters, and sugar alcohol borate esters (sorbitol, mannitol) [10, 11, 18, 35, 36]. Transported from the roots to various organs of the plant, there are two types of SBEs: (a) water-soluble SBEs in the fluid sap (mainly, sugar–borate esters and sugar alcohol borate esters) and (b) structurally water-insoluble B (mainly, B-RG-II) settled in the cells. Simultaneously, and within the same tissue, these two B-containing species may assume distinctly different roles in plant development. Despite the fact that a large amount of B is found in the pectic portion of the cell’s wall, (specifically at low B concentrations), more than 60% of the B is in a soluble state [14, 21, 22, 37, 38]. The mass distribution of water-soluble vs. structural B retrieved from one sample was correlated with that of the mass of total B measured by the dry ash method. These two types of B-containing moieties yielded the following the following B concentrations: 14.1 ± 2.8 mg/kg to 90.9 ± 2.5 mg/kg, as water-soluble B, and 27.2 ± 3.2 mg/kg to 67.1 ± 1.7 mg/kg, as structural (water-insoluble) B, respectively [39]. B has been shown to be complexed to fructose borate esters (FBEs) and also to glucose borate esters (GBEs) in the extracellular nectar from peach [14, 15]. FBEs are found in various vegetables and fruits that are subsequently consumed by humans and animals [16]. Plant-based materials serve as the fundamental dietary source of organic B-containing molecules. Plant-based materials do not serve as a primary source of inorganic B-containing molecules. CaFB, the most common form of FBE, is naturally found in fresh fruits, vegetables and honey, and in dried fruits (plums, raisins, apricots) [20]. Fructoborate is found as a protonated diester fructoborate (PFB) resulting from the sorbitol metabolic pathway in the phloem and extracellular peach nectar [19, 21]. Moreover, the pH of the phloem exudate (pH 8.5) favors the development of a *bis*-sucrose borate complex having weaker stability constants of borate (compared to the higher association constants for fructose–borate compounds) [40–43].

Today, CaFB is industrially manufactured in a nature-identical form using a chemical synthesis according to Miljkovic (1999) and Hunter (2016) patents [44, 45]. This industrially produced CaFB is being widely used as a dietary supplement for joint health, specifically for modulation of the symptoms of age-related joint discomfort and degeneration [17, 19]. CaFB has been presented in numerous published papers that report its chemistry [20, 33, 46, 47]. Additionally, it has been repeatedly reported to significantly reduce pain and improve flexibility in a clinical setting as evidenced by improvements in Western Ontario and McMaster Universities Arthritis Index (WOMAC) and McGill Indices [27–30].

It is interesting that concerns related to “total B intake” persist today despite mounting evidence that different B-containing molecules clearly have varying levels of benefit and/or potential toxicity. Much if not most of all toxicological research on B has been accumulated due to animal testing after ingestion of boric acid, despite the fact that boric acid itself does not significantly contribute to the mammalian fruit and vegetable diet. This early attention to boric acid/borate toxicity was mostly due to the well-known presence of inorganic B in drinking water and a (then) relatively scant amount of knowledge related to organic B-containing molecules found in fruits and vegetables. Inorganic B can be found in water supplies in varying amounts throughout the world dependent upon the locale. These variances ultimately affect both the content of organic B-molecules in locally grown plants and the amount of exposure to inorganic B-molecules ingested from local drinking water. However, as will be presented here, inorganic B-containing molecules manifest rather different safety profiles than the organic B-containing molecules (the SBEs) found in fruits and vegetables.

As an illustrative example of this thinking, the National Academy of Sciences/The Institute of Medicine (NAS/IOM) Panel on Micronutrients revealed that after taking into consideration all of the potential dietary “B sources,” the approximate intake per person/day might be as high as 5 mg [48–50]. However, due to the Western diet shift from plant to animal foods and because of the highly processed nature of the American diet, these levels may be lower, perhaps in the range of 0.5 to 3 mg, with 1 mg as an average. In most European countries, total B intake from natural sources is currently thought to be in the range of 2 to 10 mg per day [50]. In France, for instance, the B intake has been reported between 5 and 18 mg, due to ingestion of high quantities of wine. However, irrespective of whether or not the aforementioned figures are accurate, it is important to note that the prevailing current attention is clearly focused on total B intake rather than on assessment of relative intake of distinct B-containing molecules. The following Table 1 shows a potentially more useful comparison of total B and fructose borate ester contents of various foods.

**Table 1:** Total content of boron and fructoborate esters of various foods

Food item	Total boron (μg/g)	Fructoborate esters (μg/g)
Apple	25	3.5
Apricot	n.d.	16
Dandelion root (*Taraxaci radix*)	200	80
Figs	35	15
Flaxseed sprouts (*Lini semen*)	800	80
Honey (*Mel*)	12	7
Raisin	n.d.	79
Tomato paste	20	7

*n.d.* not determined

Depending on the plant, vegetative organ, and age of species, different amounts of boron complexing sugars (BCs) have been found [14, 22]. Most of the intake of fructoborate esters (> 90%) comes from apples, grapes, onions, and wheat. High levels of BCs (fructose and sorbitol) were measured in apple juice (4.12–6.76 and 0.11–0.51 g/100 mL, respectively) [14]. Normal human dietary intake of BCs also depends on the individual’s dietary style and the nutritional status of the ingested foods, e.g., vegetarians exhibited levels of 1.47–2.74 mg B/day (men) and 1.29–4.18 mg B/day (women) [50]. High amounts of B were highlighted for avocado, flax seeds, nutty spread, wine, raisins, peanuts, and various nuts [51].

For CaFB and related compounds, the daily average sugar–borate intake was estimated to be about 35 mg (75 mg 95th percentile intake) (based on IOM Committee’s estimation of an assumed 5 mg of daily B intake). Mathematically converted, this means that the typical daily dietary intake (from fruits and vegetables) of CaFB (containing only 2.5% B by weight) and related complexes might be as high as 165 mg/individual/day. When administering an encapsulated daily dose of 216 mg of industrially produced CaFB, the B intake (5.4 mg/day) does not exceed the safe upper limits of 6–20 mg B/day [18–20, 52], according to World Health Organization (WHO) and Organization for Economic Co-operation and Development (OECD) regulations.

In contrast to the aforementioned “safe upper limits,” a recent study in rats highlighted a no-observed-adverse-effect level (NOAEL) of 1161.3 and 1171.1 mg calcium fructoborate/kg body weight (b.w.)/day (for males and females, respectively), which translates to 29.03 mg/kg and 29.28 mg/kg b.w. B/day for male and female rats, respectively [53]. Based upon these results, and converted for comparison, this would equate to > 1.7 g daily B intake in a 60 kg human, adding some interesting dimension to any previous considerations related to B’s daily safe upper limits.

Mass spectroscopy was utilized to evaluate the existence and quantity of CaFB in serum gathered from mice gavaged with CaFB at a dosage of 650 μg per mouse for 30 and 60 min. Results demonstrated that CaFB was conveyed from the gastrointestinal (GI) tract to bloodstream unmodified and in a time-dependent manner [26, 54]. Thus, it has been confirmed that CaFB could (a) have a distinct purpose and bioactivity of its own or (b) serve as a controlled-release supply of B that does not convert back to BA in tissues and blood. This opens opportunities for further investigation into SBE metabolism and also for use of this technology for investigation into transport of sugar derivatives of other elements of nutritional importance (for example, magnesium or selenium). The biological and clinical benefits observed thus far for CaFB [17, 27, 29, 30, 33, 54, 55] suggest that B-containing sugars are indeed unique and extraordinary participants in the realm of B-containing molecules.

## Chemistry and Biochemistry

SBEs are mono- or *bis*-sugar–borate esters that include one or two monosaccharide molecules connected to a B atom [16]. Active SBEs within plants are fructose borate and glucose borate esters, *bis*-sucrose borate esters, sugar alcohol borate esters (sorbitol, mannitol), and pectic polysaccharide borate esters (RG-II) [10, 18, 36, 56].

**Fig. 1:**
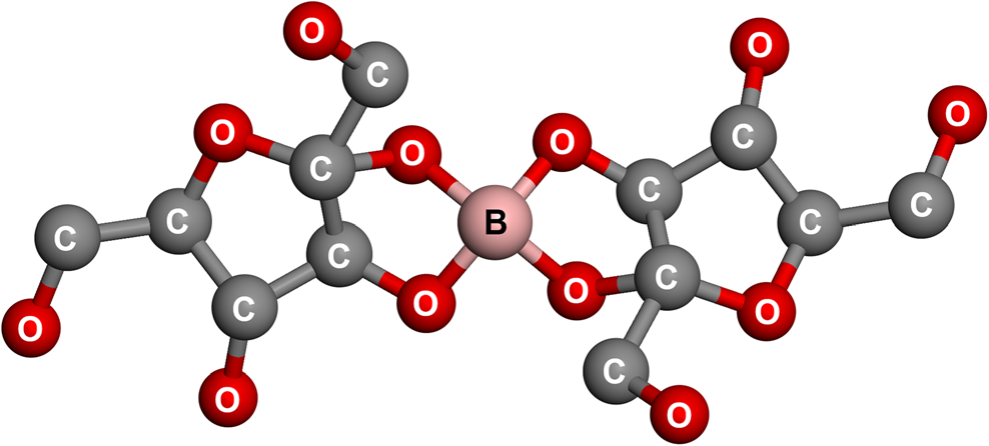
Fructoborate anion structure

The stable esters of BA are complexes in which BA forms a connection between two carbohydrate molecules, e.g., fructose-B-fructose [57, 58]. FB, an anion complex of composition (C_6_H_10_O_6_)_2_B^−^, has been identified from plants (Fig. 1) [54, 59, 60]. The investigation of this “nature–identical” complex has been performed using thermogravimetric analysis (TGA), X-ray diffraction (XRD), inductively coupled plasma–mass spectrometry (ICP-MS), Fourier-transform infrared (FTIR) spectroscopy, and Raman spectrometry techniques [46, 47, 54]. Also, using liquid- and solid-state ^11^B and ^13^C nuclear magnetic resonance (NMR) spectroscopy, the fructoborate anion structure was defined [28, 33] (Fig. 1). The fructoborate complexes in plants are water-soluble and have diastereoisomer forms (R and S), but it is still unknown which form is natural or which form possesses the biological activity [33].

**Fig. 2:**
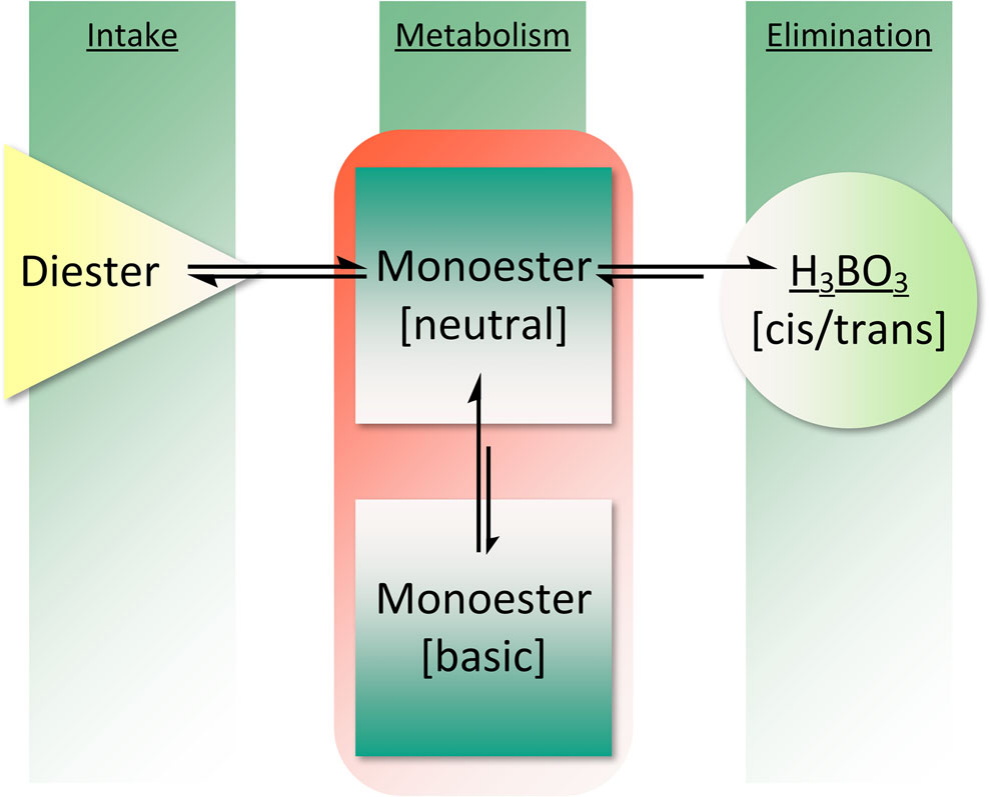
FBE speciation in the human metabolism

**Fig. 3:**
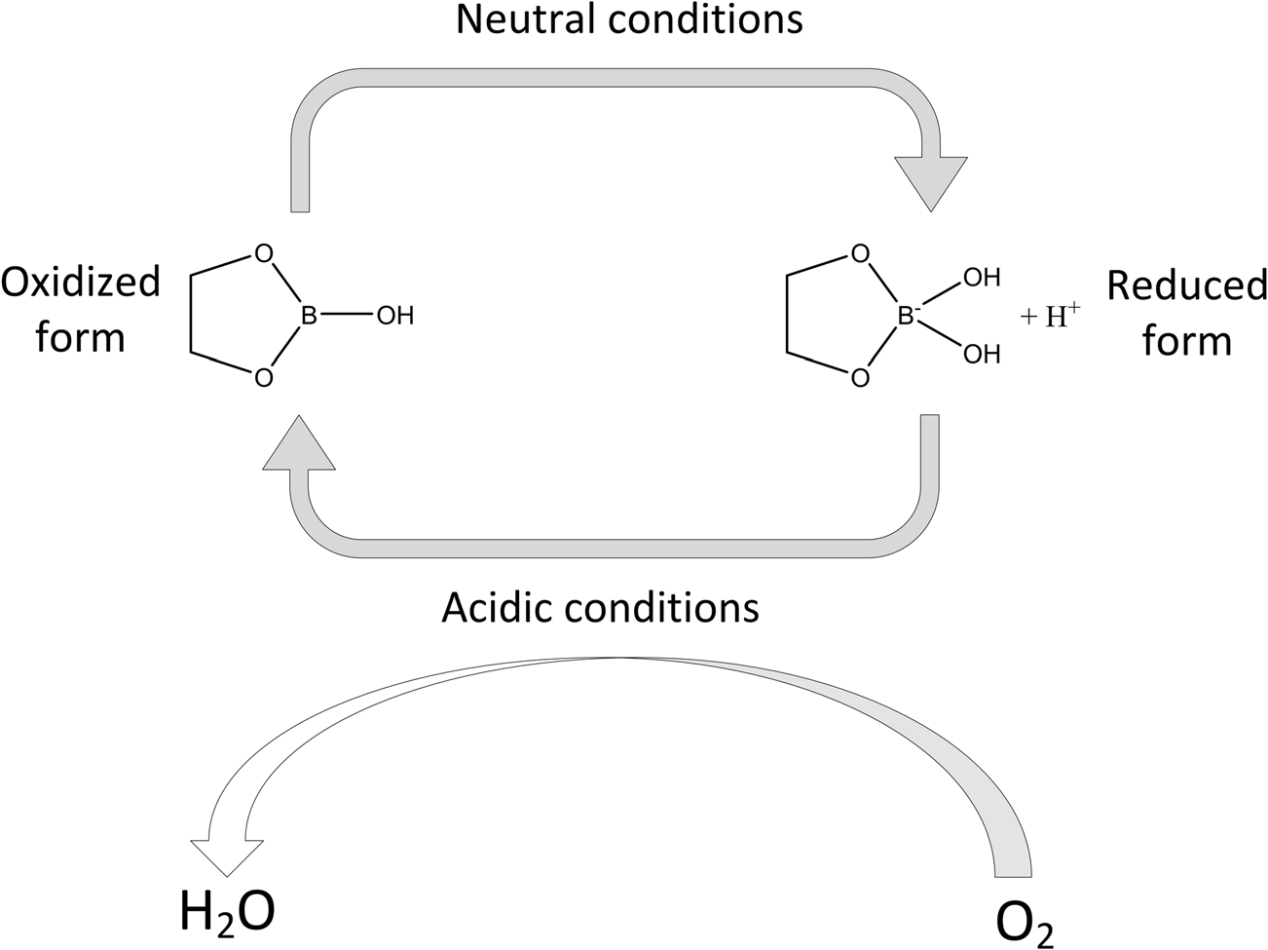
Metabolism of FBs in enzymatic system

The results of the thermal assay (TGA), combined with those of XRD, FTIR and Raman spectroscopies, high-performance thin-layer chromatography (HPTLC) [61], and liquid chromatography–multistage accurate mass spectrometry (LC-MSn) [20], led to the conclusion that an industrially produced CaFB is a similar product to the diester fructoborate anion (Fig. 1), and identical to the fructoborate molecule found in nature, including 2.5 ± 0.1% B and 4.6 ± 0.1% Ca [33]. Some studies have identified three types of B-containing molecules of CaFB in aqueous solutions: free BA, the diester, and the monoester complex (the speciation of fructoborates is highlighted in Fig. 2) [20, 33]. Subsequently, fructoborate species may possess a biological role such as functioning as a coenzyme or cofactor (Fig. 3) [25, 62]. This is an intriguing concept that could be a scope of future investigations.

## Nutritional Significance

The potential nutritional significance of SBEs in the prophylaxis of chronic diseases has been previously acknowledged in the global community [4, 16, 63]. Side effects of SBE insufficiency are non-specific and may include joint inflammation, bone loss, osteoporosis, and lower immunity [19, 64]. In some countries, borate supplementation might be helpful for prevention of osteoporosis and osteoarthritis, breast and prostate cancer, cardiovascular diseases, and, as of late, as an adjuvant for osteochondrosis in people and animals [65]. Moreover, SBEs were found not to be related to the collagen matrix but are almost completely and particularly situated inside the mineral part of bone [66]. It is suggested that SBEs influence bone mineralization by participating in the homeostatic regulation of levels of serum steroid hormones (estrogen and testosterone) and the metabolism/and usage of other mineral and calcium components of the bone [51, 67, 68]. Moreover, total B levels in the blood and bone are known to be related to health status and age and to diminish during disease states [4]. As shown in Table 2, B and its species play significant nutritional and biological roles in plants and consequently humans and animals.

**Table 2:** Fructoborate esters in plant-based foods and in dietary supplements

Sources	Salts of diester fructoborates (such as calcium, magnesium, zinc) are being stored in dried fruits and probably in fruits, nuts, vegetables, whole grains, and honey too [19, 20].
Dietary supplements	CaFB is found on the market as FruiteX-B® (commercial name). It is also found in multi-vitamin and multi-mineral–vitamin as ingredient in various formulations.
Absorption	Fructoborates are absorbed in the small intestine. Our bodies have no capability to synthesize them from fructose and boric acid. [69].
Interactions	Fructoborates have positive synergic interactions with calcium, magnesium, zinc, iron, and copper. The amount of fructoborate should be increased when the amount of iodine, fluoride, and silicon from the body increases as well.
Antagonized by	Fructoborates are antagonized by silicon as orthosilicic acid [66], by iodine [70], and by fluoride [71].
Storage	Fructoborates have been found in mice (bone, heart, brain, liver, and muscle) [69].
Excretion	In animal metabolism (mice), fructoborates are metabolized initially in fructoboric acid and then in boric acid and excreted mainly through urine [69].

The physiological influence of dietary SBEs is typically more pronounced during concurrent nutritional insult, especially vitamin D deficiency [16, 63]. In humans, dietary boron influences serum calcitonin and Ca^2+^ concentrations as well [67]. The mechanism(s) through which B as SBE modulates mineral metabolism in a variety of tissues, especially during concurrent vitamin D deficiency, is under active investigation. Low SBEs in the diet have been reported to be associated with increased serum triglyceride concentrations [16]. The potential relationship of B, insulin secretion, and plasma glucose concentrations is also under active investigation. Vitamin supplements for animal diets typically contribute inconsequential amounts of B to the total diet [72], and in general, B is formulated within said animal supplements in an inorganic form (i.e., BA) or a non-naturally occurring form (i.e., sodium tetraborate or boron citrate). This may be of particular human food chain significance as related to industrially cultivated herbivores that would most naturally ingest organic SBEs via grazing.

The most recent research shows that in bacteria the B signaling molecule AI-2 (furanosyl borate diester) can contribute to host health (gut flora) and to protection from pathogens [73]. We hypothesize that since SBEs are similar to the AI-2, they may also promote probiotic capabilities in some bacteria and reduce virulence in others. Further research is required to confirm this hypothesis.

## Toxicology

### Cytotoxicity

Zinc fructoborate (ZnFB), a novel zinc–boron complex, has a low cytotoxic effect on the normal Vero cell line. PFB and ZnFB were tested for the inhibitory effect on the Vero kidney cell line at different levels of concentration [69]. Cytotoxicity was measured using the 3-(4,5-dimethylthiazol-2-yl)-2,5-diphenyltetrazolium bromide (MTT) assay. The compounds exhibited a low cytotoxic effect on the cell line. The cell culture was more resistant to PFB than to ZnFB. For PFB, cell viability is affected above the minimal toxic concentration of 3 mM. For ZnFB, cell culture was affected both from a morphological and viability point of view (even more than the positive cytotoxicity control) above the 0.5 mM toxic concentration. ZnFB did not affect cell viability at doses ranging from 0.01 to 0.1 mM.

### In Vivo Toxicity

In vivo, SBEs have been shown to be less toxic than other B-based complexes, such as BA, inorganic borates, boronates, or boranes [16]. CaFB has a median lethal dose of 18.75 g/kg b.w. (0.525 g elemental B), compared with 2.6 g/kg b.w. for BA (0.462 g elemental B) [26, 54]. Moreover, using standard assays for bacterial and mammalian systems, sub-chronic (28-day) and chronic (90-day) dietary administration of CaFB did not exhibit genotoxicity or mutagenicity. The mean levels of CaFB tested in rats were 386, 775, and 1161 mg/kg b.w./day (for males) and 392, 784, and 1171 mg/kg b.w./day (for females), with no symptoms of toxicity, histopathological changes or fatalities [53]. As mentioned earlier, during this 90-day toxicology study on rats, the highest dose of CaFB administered with no signs of toxicity was 1171 mg/kg b.w. This is equivalent to 104,517 mg CaFB (> 1700 mg elemental B) for a 60-kg human [26, 53]. Based upon this evidence, and directly pertaining to the aforementioned limits defined by well-intentioned health organizations and government agencies regarding potential “boron toxicity” concerns, these authors respectfully argue that the limits related to boron toxicity are now in fact misinformed, archaic, and overly narrow since much if not all of the data used to define such levels have been based upon previous testing that was conducted using non-dietary, inorganic B-containing compounds (BA). Said limits may be biologically applicable to inorganic BCCs in local water supplies but, according to current evidence, should not be applied to organic SBEs (or their nature-identical equivalents) found in fruits and vegetables.

## Clinical Evidence

### Joint Degeneration and Discomfort

During an initial clinical study organized at the Orthopedic Clinic of the University of Novi Sad, Serbia, 20 osteoarthritis (OA) patients were split into two groups: (a) subjects with mild and medium OA (group A) and (b) subjects with severe OA (group B). CaFB was administered to group A, in doses of CaFB standardized to 6 mg of B, while group B received doses of CaFB standardized to 12 mg/day of B (two 6 mg doses). Mobility and flexibility returned to 50% of the patients during the first month and to 62.5% by the end of the second month. These early results strongly suggested that CaFB could be effective in reducing joint discomfort and stiffness as well as in helping to reduce the need for use of non-steroidal anti-inflammatory drugs (NSAIDs) [26].

Another double-blind placebo-controlled pilot study reported that a twice-daily 108 mg dose of CaFB significantly improved knee discomfort during a 2-week period when compared to placebo [27].

A subsequent 14-day double-blind placebo-controlled comparative study was conducted on 60 participants with self-reported knee discomfort. Study subjects were randomized into two groups, receiving either a twice-daily dose of 110 mg CaFB or placebo. Supplementation with CaFB significantly improved knee discomfort in the study subjects. Significant reductions of mean within-subject change in WOMAC and McGill Pain Questionnaire (MPQ) scores were observed for the CaFB group, compared to the placebo group, at both 7 and 14 days after treatment. The CaFB group showed greater improvement at both 7 and 14 days. In comparison, the placebo group did not exhibit any change in WOMAC and MPQ scores [29].

Also a double-blind, placebo-controlled randomized comparative 14-day study was conducted in order to compare and evaluate the effects on subjects with self-reported joint discomfort following treatment with (a) a blend of glucosamine and chondroitin sulfate; (b) a blend of glucosamine, chondroitin sulfate, and CaFB; or (c) a placebo. Treatment with glucosamine combined with chondroitin sulfate and CaFB resulted in a statistically significant 24% reduction of mean WOMAC score and a 25% reduction of mean McGill index at day 14 over baseline. Under these experimental conditions, results showed that short-term treatment with glucosamine and chondroitin could be efficacious only if used in combination with CaFB [28].

Most recently, a longer-term (90-day) double-blind, placebo-controlled randomized study was conducted in order to measure effects of CaFB supplementation on knee discomfort. In this study, the effects of once-daily and twice-daily dosing of CaFB on knee joint discomfort for 90 days were compared. One hundred and twenty subjects with self-reported knee discomfort were randomized into three groups (each group, *n* = 40). The study groups received: (a) 108 mg CaFB twice per day (group 1), (b) 216 mg CaFB in a single dose (group 2), or (c) placebo. There were no significant observed differences between group 1 and group 2 results. No changes were observed in the WOMAC and MPQ scores in the placebo group. Both CaFB groups showed significant early and continuously improving levels of knee comfort for the duration of the study. Knee comfort continued to significantly improve throughout the duration of this 90-day study. No significant differences were observed between the once-daily and the twice-daily doses of CaFB, thereby supporting use of a single daily dose [30].

### Anti-inflammatory Processes

CaFB has a beneficial effect on the various anti-inflammatory processes: (a) it regulates macrophage production of inflammatory mediators, (b) it suppresses cytokine production, and (c) it inhibits the evolution of diseases associated with endotoxins [74]. Various tests, both on humans and animals, with CaFB doses that delivered standardized amounts of B that ranged between 1 and 7 mg (0.025–0.175 mg B)/kg b.w./day resulted in a strong anti-inflammatory action [17, 54, 64]. Also, no side effects were observed on humans. CaFB has been shown to have similar effect of ω-3 fatty acids in animal model [72]. The anti-inflammatory action of CaFB might be the result of the modulation of serine proteases discharged by inflammation-activated leukocytes: (i) by the modulated leukotriene synthesis and (ii) by the decrease of reactive oxygen species (ROS) created during neutrophils’ respiratory burst. In addition, CaFB may modulate the synthesis of eicosanoids (proinflammatory prostaglandins) derived from arachidonic acid (AA). It has been demonstrated that high B administration leads to its inclusion in the phospholipids in the cell membrane, somehow replacing the AA-derived eicosanoids and thus building up ω-3-derived eicosapentaenoic acid [75]. In vitro studies also show that CaFB has a serious effect, depending on its concentration, on the human polymorphonuclear (PMN) leukocyte cells. Therefore, a 24-h treatment with CaFB on *N*-formylmethionyl-leucyl-phenylalanine (fMLP)-stimulated PMNs ensured a dose-associated decrease of the respiratory burst. The reduction of ROS level was noticeable even when non-cytotoxic concentrations were used (e.g., by half at 450 μM CaFB). This emphasizes the strong possibility that CaFB is a superoxide ion scavenger and may have anti-inflammatory activity [25, 74]. It is reasonable to consider that the CaFB molecule or its borate residue could modulate the stimulation of the enzymatic complex of nicotinamide adenine dinucleotide phosphate (NADPH)-oxidase and thereby restrict the molecular oxygen from producing superoxide anion. An increase in Ca^2+^, interceded via the binding of fMLP to its receptor, has been displayed as a part of a signaling cascade that stimulates the plasma membrane-localized oxidase [74]. A relatively recent study demonstrated that the treatment of lipopolysaccharide (LPS)-stimulated RAW264.7 macrophage cells with CaFB achieved an inhibition of interleukin (IL)-1β, IL-6, and nitric oxide (NO) release in the cell culture media, a generation boost of tumor necrosis factor-alpha (TNF-α), and had no impact on the expression of LPS-induced cyclooxygenase-2 (COX-2) protein [75]. Even though these measurements are not currently included in the routine assessment of clinical cardiovascular risk, numerous pro-inflammatory markers have been highly correlated with the development of cardiovascular disease. One of the most interesting is the monocyte chemoattractant protein-1 (MCP-1) [76]. MCP-1 is generated by different types of cells within the arterial wall, and various substances can induce its expression including cytokines, minimally modified low-density lipoproteins (LDLs), homocysteine, and shear stress, as well as other factors [77]. MCP-1 expression-induced macrophages boost atherosclerosis evolution [76]. In humans, high MCP-1 plasma levels are associated with the severity of cardiovascular issues. Some researchers have theorized that inhibiting or decreasing the MCP-1 expression might be useful in countering the development of unhealthy heart conditions [78]. Moreover, the cytokines IL-1β and IL-6 provide hints to the existence of inflammatory processes. The systemic rise of these inflammatory molecules, along with TNF-α and CRP, develops during the process of atherosclerosis. IL-1β upregulates atherogenesis by contributing to the inflammation of the arterial wall, leukocyte chemotaxis/adhesion, and the rupture of atherosclerotic plaques [78]. Therefore, serum levels of IL-6 alone or in combination with other biological markers are potential triggers for coronary pathologies. A reduction of blood levels of these inflammatory cytokines (IL-1β, IL-6, MCP-1) was reported in a recent in vivo study on CaFB [64]. All results suggest that CaFB could provide beneficial support to a healthy cardiovascular system by positively affecting the blood levels of these markers [4].

Other recently published clinical research has demonstrated the capacity of CaFB to regulate essential markers that are associated with the body’s inflammatory response mechanism. Particularly, the said studies report that CaFB significantly regulated high CRP serum levels in humans [17, 27, 79]. High CRP levels in the blood are acknowledged as a risk indicator for cardiac diseases and also is considered to have prognostic value in coronary artery disease. As measured by high-sensitivity (hs)-CRP, results revealed that resveratrol and especially CaFB (39.7% decrease after 60 days) had favorable effects, significantly lowering the hs-CRP level. These results for CaFB are in direct accord with prior studies in which similar doses induced significant decreases in hs-CRP concentration [27, 80]. This further supports the strong anti-inflammatory effects of CaFB.

### Oxidative Stress

In addition to the reported useful effects of B-containing molecules on knee/joint function, bone density (osteoporosis), cognitive health, and prostate cancer, CaFB exhibited promising antioxidant properties that could have potential clinical applications [81, 82]. In this regard, in vitro measurement of the antioxidant activity of CaFB on the potential for healing of skin wounds was conducted on human keratinocytes cell culture as an experimental model of human skin. In was found that CaFB decreased the amount of intracellular ROS induced by exposure to oxidative stress (exogenous hydrogen peroxide) [25].

### Osteoporosis

The Department of Orthopedic Medicine at the University of Novi Sad conducted a study to examine the relationship between CaFB intake and vitamin D3 serum levels [83]. Study subjects were administered one capsule of 226 mg of CaFB daily for 60 days. The study subjects were vitamin D3-deficient at the onset of the study. The study revealed enhancement regarding the vitamin D3 metabolism following CaFB supplementation. At the end of the study, 85% of the subjects showed an increase in vitamin D3 levels in blood. Prior research results showed a connection between the deficiency of vitamin D3 and a high risk for cardiovascular disorders as well as a vitamin D3 influence on joint and bone health [17]. A practical and clinical research project regarding effects of CaFB on osteoporosis was conducted in Romania [84]. CaFB has also been reported to stimulate differentiation of osteoblasts from bone narrowing as well as to restrict superoxide formation within cells [79, 85]. Additionally, it synergizes with dexamethasone to increase the bone mineralization [85].

## Mechanisms of Action

The unique plant-based organic boron-containing compounds (BCCs) (fructoborates [FBs] and glucoborates [GBs]), could be carried by means of the sugar–active transporters, whereas they have lowered the permeability due to the unchelated borate [16]. Although B species transporters have not yet been conclusively identified in animals/humans [4], it is our hypothesis that SBEs transport in the cell could occur via sugar transporters (GLUTs) by facilitated diffusion [16], at the very least for those SBEs containing non-charged trigonal borate (Fig. 3). GLUTs may potentially be one of the most convenient avenues for introducing fructoboric acid (FBA) into cells [16].

### Non-enzymatic Metabolism: Catalyzed CO_2_ Hydration

Recent research has shown that the development of HCO_3_^−^ ion is stimulated by BCCs acting as CO_2_ hydration catalysts. It appears that the mechanism involved is analogous to the enzymatic activity of carbonic anhydrase [86]. Practically, the buffer efficiency of the HCO_3_^−^ (pH 7.4) is overtaken by the borate anion pairing with *cis*-diol esters through the appearance of some complexes with polyalcohols, sugars, and *cis*-diol organic acids (p*K*a 3.5–7.4) [4].

### Enzymatic Metabolism

#### SBEs as Pleiotropic Modulators for Cell Signaling Pathways

In addition to investigating SBEs essential association with various regulatory and signaling molecules (as well as with transcription factors), it could be of importance to confirm the connection between the borate anion/sugar–borate anion/B shortage and the Ca^2+^ influx and to fully determine the Ca^2+^-releasing systems coordinated by adenylates or inositides [4]. Additionally, adenylates or inositides, being potential targets for B [5, 87], could be a segment of the signaling pathways afflicted by B deficiency. The fact that Ca^2+^ counters the expression of most genes that are affected by the deficiency of B indicates that BCC metabolism may be linked to signaling pathways mediated by Ca^2+^. Further, it has been shown that BA can inhibit proliferation of some tumor cells by influencing the Ca^2+^ release through cyclic adenosine diphosphate ribose (cADPr). This result is based on BA’s ability to bind NAD^+^, which is the substrate for ADP-ribosyl cyclase [88]. In addition to investigating an essential connection of B with transcription factors and signaling and regulatory molecules, such as glycans or micro-ribonucleic acids (miRNAs), it would be intriguing to investigate and determine whether deficiency of B alters Ca^2+^ influx [89] and/or if B also intervenes within the Ca^2+^-releasing system that is dependent upon inositides or adenylates. Such studies would reveal new insights regarding B’s role in the regulation of gene expression and on the impact of the B–Ca^2+^ relation on gene expression.

The outcome of these investigations could establish the connection between borate anion/sugar–borate anion/B and calcium related to certain genes’ regulation and expression. Additionally, the natural BCCs found in plants (glucose– as well as fructose–borates) have been predicted to be implicated within cell signaling systems of ROS [25, 62]. Different clinical investigations have demonstrated the capacity of CaFB to regulate molecular markers of inflammation, mainly CRP [64]. Fructoborate has been recently found to be a powerful catalyst in various natural reactions in plants and perhaps it even acts as a coenzyme [62]. One study analyzed whether CaFB exerted antioxidant characteristics on cell cultures of human keratinocytes in order to determine whether CaFB could have potential application for use on skin wounds. The cells were subjected to hydrogen peroxide as a means of emulating natural oxidative stress. The results showed that CaFB reduced the generation of intracellular ROS, thereby implying that CaFB may impart a superoxide dismutase (SOD)-like effect [25].

Various researches have demonstrated that hyperhomocysteinemia is associated with a high risk of atherosclerosis, as well as with venous thromboembolism, defects of neural tube in fetus, and various pregnancy-related issues. The effect of CaFB on homocysteine levels was studied in a more extensive human clinical trial [77, 90], in which a significant reduction in homocysteine was reported. Results showed homocysteine reductions to be around 5% (from baseline, mean 94.5 ± 54.1%, middle 81.1%, territory 55.1–334.1%, *p* = 0.004) [64].

The fact that B appears in a signaling molecule in bacteria [35] and that the borate complexes can interact with regulatory proteins [7] suggests the intriguing probability that B often acts in an organic form through interaction with transcription factors, thus explaining a gene expression wide alteration [91]. B as SBEs may also act via the phosphorylation steps and may activate some kinases. Because phosphorylation activates several initiation factors, the same action of SBE on kinases could also act on translation. Finally, SBEs may serve to stabilize RNA and to regulate ribonuclease activity because it has been reported that this enzymatic activity is increased in boron-deficient plants [5]. It has been determined that B is concentrated in the cell membrane. SBEs function by creating a positive electrostatic charge in the membrane by capturing a loose electron from a donor that is disturbed by the actions of gravity, phytohormones, and light. The positive charge that is subsequently generated could control the flow of ions through the cell membrane pores. This positive charge might also attract negatively charged molecules (such as nucleic acids) and, furthermore, could facilitate, control, or initiate some vital reactions important for the division and elongation of cells, as well as in flowering in plants [92].

Subsequently, the pleiotropic activities that have been attributed to CaFB result from its complex chemistry and molecular structure and from its capacity to influence several signaling molecules such as inflammatory molecules, protein kinases, proteasome, carrier proteins, and metal ions [17]. CaFB is a functional molecule that has the ability to regulate the biological activity of various signaling molecules directly or indirectly by connecting through hydrogen bonding and covalent connections. The molecular targets affected by CaFB can be up- or downregulated and include enzymes, transcription factors, protein kinases, inflammatory mediators, cell-cycle regulatory proteins, receptors, growth factors, adhesion molecules, cytokine, and cytokine receptors. Given these targets’ diversity, it can easily be envisioned how CaFB may be a mediator of pleiotropic activity [4, 19].

The activity of SBEs on various processes in human cells is also based not only on the regulation of a variety of enzymatic activities that include cytochrome (cyt) b5 reductase, serine proteases, messenger RNA (mRNA) splicing, glutamyl transpeptidase, xanthine dehydrogenase (XDH), alcohol NAD-dehydrogenases, xanthine oxidase (XOD), alkaline phosphatase, and division of cells but also on receptor binding imitation and on apoptosis induction [63]. SBEs form complexes between the hydroxyl and amino groups in proteins and target glutamine, histidine, lysine, proline, and serine residues. Consequently, CaFB appears to be distinct from, and in many cases superior to, BA/borate due to its complex mechanisms of action, both intracellularly (free BA) and extracellularly (fructose–borate esters) [4, 19].

#### SBEs as Essential Molecules in Biological Processes (Such as Being a Conditionally Essential Nutrient/Vitamin)

**Fig. 4:**
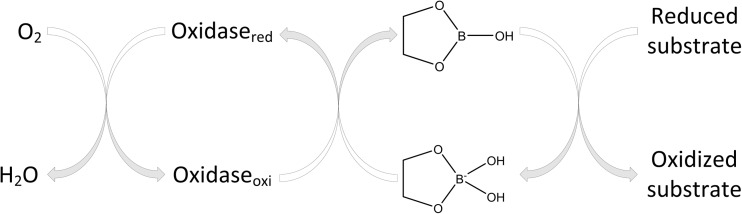
SBE bioactivity may be associated with monoester’s speciation (neutral and basic monoester)

The diversity of the SBEs’ actions is further enhanced as a result of its reactions with biological molecules that contain adenosine or are formed from precursors of adenosine. Diadenosine phosphates and S-adenosylmethionine have a higher affinity for B than any other known B ligand found in animal tissues [32]. Diadenosine phosphates exist in all animal cells and act as signal nucleotides linked to neuronal response. As shown in Fig. 4, the monoester sugar–borate may be a coenzyme for the oxidoreductase and might be influential in many disorders that can be affected by the nutritional intake of SBEs (arthritis, certain cancers, diabetes, impaired brain function, and osteoporosis).

B additionally binds the oxidized NAD^+^ and in this way could impact the reactions in which it participates [4, 16]. One of the aspects of extracellular NAD^+^ is its binding to the plasma membrane receptor CD38, which is an ADP-ribosyl cyclase that transforms NAD^+^ to cyclic ADP ribose. The cyclic ADP ribose is released intracellularly and binds to the ryanodine receptor, which then enables the release of Ca^2+^ from the endoplasmic reticulum. Researches on cell cultures demonstrate that boric acid binds to, and that it is also a reversible inhibitor of, cyclic ADP ribose. Hence, we estimate that SBEs could be biologically active by binding NAD^+^ and/or cyclic ADP ribose and by restricting the release of Ca^2+^, which is a signal ion for numerous mechanisms in which SBEs have appeared to have an impact, including release of insulin, bone development, immune reaction, and brain function. In addition, SBEs can be active through the formation of diester borate complexes with glycolipids, glycoproteins, and phosphoinositides that contain *cis*-hydroxyl groups within the membranes [4, 16]. The diester borate complex could function as a calcium chelator as well as a redox metabolism modifier that influences membrane function and stability. Diester borate complexes in membranes could control the transmembrane partitioning of B. These theories propose that SBEs could influence cell differentiation, embryogenesis, and organogenesis [4]. The fructoborate complex has been recently found to be a powerful catalyst in various biological reactions in plants, and it may act as a coenzyme (Fig. 4) [62].

## Discussions and Future Perspectives

### Prevention of Osteoporosis by Fructoborate Intake

The trace element B, formerly considered to be without nutritional value, is naturally provided as SBEs to animal species via their ingestion of plants and, to a greater or lesser degree, as inorganic B via the water supply. As such, B has a pivotal role to play in fighting against osteoporosis. B impacts human’s steroid hormone metabolism, thereby influencing estrogen and testosterone levels. It has been postulated that B interacts with steroid hormones by facilitating their hydroxylation and by protecting the steroid hormones from fast degradation. Nielsen (2014) stated that supplementation of B 3 mg/day (delivered via ingestion of boric acid) decreases the excretion of calcium and phosphorus in urine [8]. A significant rise of plasma steroid hormones was shown in humans and rats after B species supplementation. B species supplementation has been suggested to modulate the enzymatic catabolic hydroxylation thereby influencing the synthesis of vitamin D [83]. When all the evidence concerning B and Ca is considered, it is reasonable to conclude that B likely plays a favorable role in the metabolism of calcium. This is especially and clinically relevant in the prevention of osteoporosis and bone loss. Recent studies have determined that supplementation of B (as BA) has a positive action on the development and growth of ostrich tibia. A 200-mg/L of B supplementation in drinking water has been shown to be the best supplementation to boost bone development in ostrich chicks [93]. Dietary B supplements have been shown to improve serum B content in osteoporotic rats corroborated with data on bone formation and reduced bone resorption, indicating a strong therapeutic effect against osteoporosis [94]. Consequently, it is reasonable that CaFB supplements could be an important adjuvant in osteoporosis treatment [84] and in women’s health. Further research is required in this regard.

### Potential Effect of Dietary Fructoborate on the Aging Process

According to the oxidative stress theory, an imbalance of the homeostatic balance between ROS removal and ROS production is the main cause of aging [95]. Recent studies determined that even low doses of different B-containing compounds could increase the antioxidant capacity by acceleration of enzymatic activities [96–98]. There is also some evidence that B-containing compounds can restrict Maillard reactions through the formation of stable borate esters, making possible the simultaneous chemical evolution of sugar and amino acids in aqueous media (80–100 °C) [2]. Consequently, B may be involved in the inhibition of glycation in living systems [99]. When a dose of CaFB was administered to canines, a greater increase of soluble receptor for advanced glycation end products (sRAGE) occurred compared with placebo-fed canines [100]. Recent studies have also showed that higher plasma levels of sRAGE are associated with a reduced risk of coronary artery disease, hypertension, the metabolic syndrome, arthritis, and Alzheimer’s disease [101]. Advanced glycation end products (AGEs) have been studied in relation to diabetes, endothelial dysfunction, vascular diseases and aging itself [102]. It has been suggested that AGEs could be associated with numerous degenerative diseases including cardiovascular disease, dementia, diabetes, renal failure, osteoarthritis, and loss of sight [103].

Recent data show that B-containing compounds are implicated in NAD^+^ regeneration in the mitochondria [4, 9, 104] and in the protection of deoxyribonucleic acid (DNA) against damage [105]. The NAD^+^/NADH ratio is extremely important for boosting the mitochondria activity in repairing the nucleic acid, and borate has been shown to improve the NAD^+^/NADH ratio in animals [104]. Borate complex diesters are able to diminish mitochondrial toxicity due to their capacity to scavenge boric acid [69]. Some results also suggest that dietary B (as SBEs) is implicated in some yet unclear mechanism related to senescence. Both apples and chicken eggs have the highest concentration of B in the peels and shells, respectively, which are exposed to the environment. This could suggest that another biological role of B may be related to some protective function (protein turnover) [106].

It has been shown that the birth rate in communities with high B content (inorganic BCCs) in the drinking water was greater than that of the reference geographic zone and of the general population (*p* < 10^−4^). Further, the mortality rate in communities with high B content in drinking water was less than that of the reference geographic zone and of the general population (*p* < 10^−3^). No statistical difference was noted in the male/female gender ratios between the different zones (*p* = 0.45) [107, 108].

Dietary intake of the organic BCCs also provides very significant opportunities in the life extension and quality of life improvement. Encouraging to-date evidence warrants further research to maximize the application of B and SBEs in human and animal health, particularly in the areas of healthier aging and modulation of inflammatory conditions. It has become clear that absorption and subsequent utilization of inorganic BCCs and the organic BCCs, such as the SBEs (and CaFB in particular), confer decidedly distinct safety, biological, and functional benefits in vivo. Recent papers have reported that B is not transported as borate anion [109]. Therefore, B transport in the animal cell is still considered to be an unresolved question [110–112]. Consequently, additional research will be required to further substantiate the mechanism of B or BCC transport in animal cells. This research will help us to further understand their respective potentials for applications in nutrition, health, and medicine.

## Conclusions

The unique properties of SBEs suggest new pathways and opportunities to employ these molecules as supplemental and therapeutic agents. The recent discoveries that SBEs mimic enzymatic pleiotropic modulators or cofactors provide a key consideration in our determination that some of these B species are essential or at least conditionally essential for humans [4, 11, 68, 113]. Based upon the evidence thus far, we propose here that the safety, functionality, and efficacy of SBEs are favorable for many physiological functions when compared to inorganic boron derivatives such as boric acid/borax. This is likely attributable to the tendency of organic boron compounds to remain in their organic form (in comparison to inorganic forms that dissociate rapidly), thereby conferring benefits such as coenzyme or cofactor activities and/or anti-inflammatory effects that subsequently impart the clinically relevant benefits that have been reported [4]. Consequently, the aim of our research going forward is to more fully understand the critical role and mechanisms of action of these SBEs and their related B species in human health and, ultimately, to address following questions: (i) Is fructoborate an essential, or at least a conditionally essential, nutrient for human health, and/or (ii) could any of the aforementioned SBEs actually have sufficient importance to be eventually considered to be natural boron-containing vitamins?

We applaud the previous and ongoing efforts of fellow scientists who have so passionately contributed to the advancement of B-related research and knowledge; we militantly challenge the current misconceptions related to B toxicity; finally, we encourage the world to recognize the clearly important human and animal health benefits inherent in these various B-containing molecules, benefits that have been hiding in plain sight within our plant-based diets since the very beginning of time.

## References

[1] Templeton DM, Ariese F, Cornelis R, Danielsson LG, Muntau H, van Leeuwen HP, Lobinski R (2000). Guidelines for terms related to chemical speciation and fractionation of elements. Definitions, structural aspects, and methodological approaches (IUPAC Recommendations 2000). Pure Appl Chem.

[2] Scorei R (2012). Is boron a prebiotic element? A mini-review of the essentiality of boron for the appearance of life on Earth. Orig Life Evol Biosph.

[3] Kim HJ, Furukawa Y, Kakegawa T, Bita A, Scorei R, Benner SA (2016). Evaporite borate-containing mineral ensembles make phosphate available and regiospecifically phosphorylate ribonucleosides: borate as a multifaceted problem solver in prebiotic chemistry. Angew Chemie Int Ed Engl.

[4] Donoiu I, Militaru C, Obleagă O, Hunter JM, Neamţu J, Biţă A, Scorei IR, Rogoveanu OC (2018). Effects of boron-containing compounds on cardiovascular disease risk factors—a review. J Trace Elem Med Biol.

[5] Bolaños L, Lukaszewski K, Bonilla I, Blevins D (2004). Why boron?. Plant Physiol Biochem.

[6] Minchin PEH, Thorp TG, Boldingh HL, Gould N, Cooney JM, Negm FB, Focht E, Arpaia ML, Hu H, Brown P (2012). A possible mechanism for phloem transport of boron in ‘Hass’ avocado (*Persea americana* Mill.) trees. J Hortic Sci Biotechnol.

[7] Goldbach HE, Wimmer MA (2007). Boron in plants and animals: is there a role beyond cell-wall structure?. J Plant Nutr Soil Sci.

[8] Nielsen FH (2014). Update on human health effects of boron. J Trace Elem Med Biol.

[9] Khaliq H, Juming Z, Ke-Mei P (2018). The physiological role of boron on health. Biol Trace Elem Res.

[10] Dembitsky VM, Smoum R, Al-Quntar AA, Ali HA, Pergament I, Srebnik M (2002). Natural occurrence of boron-containing compounds in plants, algae and microorganisms. Plant Sci.

[11] Dembitsky VM, Gloriozova TA (2017). Naturally occurring boron containing compounds and their biological activities. J Nat Prod Resour.

[12] Uluisik I, Karakaya HC, Koc A (2018). The importance of boron in biological systems. J Trace Elem Med Biol.

[13] Zumreoglu-Karan B, Kose DA (2015). Boric acid: a simple molecule of physiologic, therapeutic and prebiotic significance. Pure Appl Chem.

[14] Hu H, Penn SG, Lebrilla CB, Brown PH (1997). Isolation and characterization of soluble boron complexes in higher plants. The mechanism of phloem mobility of boron. Plant Physiol.

[15] Brown PH, Shelp BJ (1997). Boron mobility in plants. Plant Soil.

[16] Scorei RI, Popa R (2013). Sugar-borate esters—potential chemical agents in prostate cancer chemoprevention. Anti Cancer Agents Med Chem.

[17] Scorei ID, Scorei RI (2013). Calcium fructoborate helps control inflammation associated with diminished bone health. Biol Trace Elem Res.

[18] Dinca L, Scorei R (2013). Boron in human nutrition and its regulations use. J Nutr Ther.

[19] Mogoşanu GD, Biţă A, Bejenaru LE, Bejenaru C, Croitoru O, Rău G, Rogoveanu OC, Florescu DN, Neamţu J, Scorei ID, Scorei RI (2016). Calcium fructoborate for bone and cardiovascular health. Biol Trace Elem Res.

[20] Xia X, Chang JS, Hunter JM, Nemzer BV (2017). Identification and quantification of fructoborate ester complex using liquid chromatography coupled with Q exactive orbitrap mass spectrometry. J Food Res.

[21] Brown PH, Hu H (1996). Phloem mobility of boron is species dependent: evidence for phloem mobility in sorbitol-rich species. Ann Bot.

[22] Brown PH, Hu H (1998). Phloem boron mobility in diverse plant species. Bot Acta.

[23] Kobayashi M, Matoh T, Azuma J (1996). Two chains of rhamnogalacturonan II are cross-linked by borate-diol ester bonds in higher plant cell walls. Plant Physiol.

[24] Brown PH, Bellaloui N, Wimmer MA, Bassil ES, Ruiz J, Hu H, Pfeffer H, Dannel F, Römheld V (2002). Boron in plant biology. Plant Biol (Stuttg).

[25] Scorei R, Cimpoiaşu VM, Iordachescu D (2005). In vitro evaluation of the antioxidant activity of calcium fructoborate. Biol Trace Elem Res.

[26] Miljkovic D, Scorei RI, Cimpoiaşu VM, Scorei ID (2009). Calcium fructoborate: plant-based dietary boron for human nutrition. J Diet Suppl.

[27] Reyes-Izquierdo T, Nemzer B, Gonzalez AE, Zhou Q, Argumedo R, Shu C, Pietrzkowski Z (2012). Short-term intake of calcium fructoborate improves WOMAC and McGill scores and beneficially modulates biomarkers associated with knee osteoarthritis: a pilot clinical double-blinded placebo-controlled study. Am J Biomed Sci.

[28] Reyes-Izquierdo T, Phelan MJ, Keller R, Shu C, Argumedo R, Pietrzkowski Z (2014). Short-term efficacy of a combination of glucosamine and chondroitin sulfate compared to a combination of glucosamine, chondroitin sulfate and calcium fructoborate (CFB) on improvement of knee discomfort conditions in healthy subjects. A comparative, double-blind, placebo controlled acute clinical study. J Aging Res Clin Pract.

[29] Pietrzkowski Z, Phelan MJ, Keller R, Shu C, Argumedo R, Reyes-Izquierdo T (2014). Short-term efficacy of calcium fructoborate on subjects with knee discomfort: a comparative, double-blind, placebo-controlled clinical study. Clin Interv Aging.

[30] Pietrzkowski Z, Roldán Mercado-Sesma A, Argumedo R, Cervantes M, Nemzer B, Reyes-Izquierdo T (2018). Effects of once-daily *versus* twice daily dosing of calcium fructoborate on knee discomfort. A 90 day, double-blind, placebo controlled randomized clinical study. J Aging Res Clin Pract.

[31] Nielsen FH (2017). Historical and recent aspects of boron in human and animal health. J Boron.

[32] Nielsen FH (2008). Is boron nutritionally relevant?. Nutr Rev.

[33] Edwards JC, Hunter JM, Nemzer BV (2014). Multinuclear NMR of calcium fructoborate complex—structure, stability, and quantitation in the presence of other ingredients, excipients or adulterants. J Food Res.

[34] O’Neill MA, Ishii T, Albersheim P, Darvill AG (2004). Rhamnogalacturonan II: structure and function of a borate cross-linked cell wall pectic polysaccharide. Annu Rev Plant Biol.

[35] Chen X, Schauder S, Potier N, Van Dorsselaer A, Pelczer I, Bassler BL, Hughson FM (2002). Structural identification of a bacterial quorum-sensing signal containing boron. Nature.

[36] Řezanka T, Sigler K (2008). Biologically active compounds of semi-metals. Phytochemistry.

[37] Pfeffer H, Dannel F, Römheld V, Bell RW, Rerkasem B (1997). Compartmentation of boron in roots and its translocation to the shoot of sunflower as affected by short term changes in boron supply. Boron in soils and plants.

[38] Matoh T (1997). Boron in plant cell walls. Plant Soil.

[39] Sun A, Xu Q, Ren L, Cao G, Gou D (2017). Non-equilibrium ultrasound-assisted solid–liquid extraction of boron present in different phases within plants by ICP-OES. RSC Adv.

[40] Verchere JF, Hlaibi M (1987). Stability constants of borate complexes of oligosaccharides. Polyhedron.

[41] Bieleski RL, Redgwell RJ (1980). Sorbitol metabolism in nectaries from flowers of *Rosaceae*. Aust J Plant Physiol.

[42] Moing A, Carbonne F, Rashad MH, Gaudillère JP (1992). Carbon fluxes in mature peach leaves. Plant Physiol.

[43] Miller EP, Wub Y, Carrano CJ (2016). Boron uptake, localization, and speciation in marine brown algae. Metallomics.

[44] Miljkovic D (1999) Boron carbohydrate complexes and uses thereof. US Patent # 5962049 A. https://patentimages.storage.googleapis.com/86/c0/28/63391aac18daf8/US5962049.pdf

[45] Hunter JM (2015) Compositions and methods for borocarbohydrate complexes. US Patent # 9102700 B1. https://patentimages.storage.googleapis.com/2c/fa/76/6abbd68b8ba311/US9102700.pdf

[46] Dumitru MD, Miljkovic D, Scorei RI, Rotaru P (2010). FT-IR and Raman spectroscopic analysis of a calcium fructoborate sample. Physics AUC.

[47] Rotaru P, Scorei R, Hărăbor A, Dumitru MD (2010). Thermal analysis of a calcium fructoborate sample. Thermochim Acta.

[48] National Academy of Sciences, Institute of Medicine, Food and Nutrition Board (2001) Dietary reference intakes for vitamin A, vitamin K, arsenic, boron, chromium, copper, iodine, iron, manganese, molybdenum, nickel, silicon, vanadium, and zinc. A Report of the Panel on Micronutrients, Subcommittees on Upper Reference Levels of Nutrients and of Interpretation and Uses of Dietary Reference Intakes, and the Standing Committee on the Scientific Evaluation of Dietary Reference Intakes, National Academy Press, Washington, DC. http://www.ncbi.nlm.nih.gov/books/NBK222310/

[49] Penn SG, Hu H, Brown PH, Lebrilla CB (1997). Direct analysis of sugar alcohol borate complexes in plant extracts by matrix-assisted laser desorption/ionization Fourier transform mass spectrometry. Anal Chem.

[50] Rainey CJ, Nyquist LA, Christensen RE, Strong PL, Culver BD, Coughlin JR (1999). Daily boron intake from the American diet. J Am Diet Assoc.

[51] Hunt CD (2012). Dietary boron: progress in establishing essential roles in human physiology. J Trace Elem Med Biol.

[52] Hathcock JN (2014). Vitamin and mineral safety.

[53] Marone PA, Heimbach JT, Nemzer B, Hunter JM (2016). Subchronic and genetic safety evaluation of a calcium fructoborate in rats. Food Chem Toxicol.

[54] Scorei RI, Rotaru P (2011). Calcium fructoborate—potential anti-inflammatory agent. Biol Trace Elem Res.

[55] Basoglu A, Baspinar N, Ozturk AS, Akalin PP (2011). Effects of long-term boron administrations on high-energy diet-induced obesity in rabbits: NMR-based metabonomic evaluation. J Anim Vet Adv.

[56] Řezanka T, Sigler K (2008). Biologically active compounds of semi-metals. Stud Nat Prod Chem.

[57] Matsunaga T, Nagata T (1995). In vivo ^11^B NMR observation of plant tissue. Anal Sci.

[58] Woods WG (1996). Review of possible boron speciation relating to its essentiality. J Trace Elem Exp Med.

[59] Wagner CC, Baran EJ (2008). Easy synthesis of CaB_2_O_4_ via pyrolysis of calcium fructoborate. Mater Res.

[60] Wagner CC, Ferraresi Curotto V, Pis Diez R, Baran EJ (2008). Experimental and theoretical studies of calcium fructoborate. Biol Trace Elem Res.

[61] Bita A, Mogosanu GD, Bejenaru LE, Oancea CN, Bejenaru C, Croitoru O, Rau G, Neamtu J, Scorei ID, Scorei IR, Hunter J, Evers B, Nemzer B, Anghelina F, Rogoveanu OC (2017). Simultaneous quantitation of boric acid and calcium fructoborate in dietary supplements by HPTLC-densitometry. Anal Sci.

[62] Cheng CY, Liao CI, Lin SF (2015). Borate-fructose complex: a novel mediator for laccase and its new function for fructose determination. FEBS Lett.

[63] Scorei RI, Popa R (2010). Boron-containing compounds as preventive and chemotherapeutic agents for cancer. Anti Cancer Agents Med Chem.

[64] Rogoveanu OC, Mogoşanu GD, Bejenaru C, Bejenaru LE, Croitoru O, Neamţu J, Pietrzkowski Z, Reyes-Izquierdo T, Biţă A, Scorei ID, Scorei RI (2015). Effects of calcium fructoborate on levels of C-reactive protein, total cholesterol, low-density lipoprotein, triglycerides, IL-1β, IL-6, and MCP-1: a double-blind, placebo-controlled clinical study. Biol Trace Elem Res.

[65] Johnson EW (2012) Prevention and treatment of osteochondrosis in animals and humans. US Patent # 2012/0328715 A1. https://patentimages.storage.googleapis.com/5b/40/16/4df6497ede2bed/US20120328715A1.pdf

[66] Jugdaohsingh R, Pedro LD, Watson A, Powell JJ (2015). Silicon and boron differ in their localization and loading in bone. Bone Rep.

[67] Devirian TA, Volpe SL (2003). The physiological effects of dietary boron. Crit Rev Food Sci Nutr.

[68] Soriano-Ursúa MA, Das BC, Trujillo-Ferrara JG (2014). Boron-containing compounds: chemico-biological properties and expanding medicinal potential in prevention, diagnosis and therapy. Expert Opin Ther Pat.

[69] Scorei IR (2018) Novel active zinc and boron-based dietary supplements for longevity and healthy life. Grant of the Romanian National Authority for Scientific Research and Innovation, CNCS/CCCDI–UEFISCDI, Project No. PN-III-P2-2.1-PED-2016-0410. http://naturalresearch.ro/?page_id=34

[70] Popova EV, Tinkov AA, Ajsuvakova OP, Skalnaya MG, Skalny AV (2017). Boron—a potential goiterogen?. Med Hypotheses.

[71] Pahl MV, Culver BD, Vaziri ND (2005). Boron and the kidney. J Ren Nutr.

[72] Criste RD, Grossu DV, Scorei R, Duca RC, Mitrut M, Ciurescu G (2005). New investigations on the effect of the dietary boron on broilers and layers; boron and food quality. Arch Zootech.

[73] Thompson JA, Oliveira RA, Xavier KB (2016). Chemical conversations in the gut microbiota. Gut Microbes.

[74] Scorei R, Ciubar R, Iancu C, Mitran V, Cimpean A, Iordachescu D (2007). In vitro effects of calcium fructoborate on fMLP-stimulated human neutrophil granulocytes. Biol Trace Elem Res.

[75] Scorei RI, Ciofrangeanu C, Ion R, Cimpean A, Galateanu B, Mitran V, Iordachescu D (2010). In vitro effects of calcium fructoborate upon production of inflammatory mediators by LPS-stimulated RAW 264.7 macrophages. Biol Trace Elem Res.

[76] Deshmane SL, Kremlev S, Amini S, Sawaya BE (2009). Monocyte chemoattractant protein-1 (MCP-1): an overview. J Interf Cytokine Res.

[77] Cheung GT, Siow YL, O K (2008). Homocysteine stimulates monocyte chemoattractant protein-1 expression in mesangial cells via NF-kappaB activation. Can J Physiol Pharmacol.

[78] Kolattukudy PE, Niu J (2012). Inflammation, endoplasmic reticulum stress, autophagy, and the monocyte chemoattractant protein-1/CCR2 pathway. Circ Res.

[79] Scorei R, Mitrut P, Petrisor I, Scorei I (2011). A double-blind, placebo-controlled pilot study to evaluate the effect of calcium fructoborate on systemic inflammation and dyslipidemia markers for middle-aged people with primary osteoarthritis. Biol Trace Elem Res.

[80] Militaru C, Donoiu I, Craciun A, Scorei ID, Bulearca AM, Scorei RI (2013). Oral resveratrol and calcium fructoborate supplementation in subjects with stable angina pectoris: effects on lipid profiles, inflammation markers, and quality of life. Nutrition.

[81] Pawa S, Ali S (2006). Boron ameliorates fulminant hepatic failure by counteracting the changes associated with the oxidative stress. Chem Biol Interact.

[82] Taranu I, Marin DE, Manda G, Motiu M, Neagoe I, Tabuc C, Stancu M, Olteanu M (2011). Assessment of the potential of a boron-fructose additive in counteracting the toxic effect of *Fusarium* mycotoxins. Br J Nutr.

[83] Miljkovic D, Miljkovic N, McCarty MF (2004). Up-regulatory impact of boron on vitamin D function—does it reflect inhibition of 24-hydroxylase?. Med Hypotheses.

[84] Ghivercea V, Grecu D, Lichiardopol C, Maria R (2004). The treatment of osteoporosis with calcium fructoborate. J Orthop Traumatol (Bucharest).

[85] Manda D, Popa O, Vladoiu S, Dumitrache C (2009). Calcium fructoborate effect on osteoblast mineralization in vitro. Bone.

[86] Verma M, Deshpande PA (2017). Computational insights into biomimetic CO_2_ hydration activities of (poly)borate ions. J Phys Chem C.

[87] Ralston NVC, Hunt CD (2001). Diadenosine phosphates and S-adenosylmethionine: novel boron binding biomolecules detected by capillary electrophoresis. Biochim Biophys Acta.

[88] Barranco WT, Kim DH, Stella SL, Eckhert CD (2009). Boric acid inhibits stored Ca^2+^ release in DU-145 prostate cancer cells. Cell Biol Toxicol.

[89] Koshiba T, Kobayashi M, Ishihara A, Matoh T (2010). Boron nutrition of cultured tobacco BY-2 cells. VI. Calcium is involved in early responses to boron deprivation. Plant Cell Physiol.

[90] Clarke R, Daly L, Robinson K, Naughten E, Cahalane S, Fowler B, Graham I (1991). Hyperhomocysteinemia: an independent risk factor for vascular disease. N Engl J Med.

[91] González-Fontes A, Rexach J, Navarro-Gochicoa MT, Herrera-Rodríguez MB, Beato VM, Maldonado JM, Camacho-Cristóbal JJ (2008). Is boron involved solely in structural roles in vascular plants?. Plant Signal Behav.

[92] Tanada T (1995). Boron as a transducer in some physiological processes of plants. J Plant Nutr.

[93] Cheng J, Peng K, Jin E, Zhang Y, Liu Y, Zhang N, Song H, Liu H, Tang Z (2011). Effect of additional boron on tibias of African ostrich chicks. Biol Trace Elem Res.

[94] Samman S, Foster M, Hunter D, Hosmane NS (2012). Part II—boron for living: health and nutrition. The role of boron in human nutrition and metabolism. Boron science: new technologies and applications.

[95] Trifunovic A, Larsson NG (2008). Mitochondrial dysfunction as a cause of ageing. J Intern Med.

[96] Türkez H, Geyikoğlu F, Tatar A, Keleş S, Özkan A (2007). Effects of some boron compounds on peripheral human blood. Z Naturforsch C.

[97] Türkez H, Geyikoğlu F, Çolak S (2011). The protective effect of boric acid on aluminum-induced hepatotoxicity and genotoxicity in rats. Turk J Biol.

[98] Turkez H, Geyikoglu F (2010). Boric acid: a potential chemoprotective agent against aflatoxin b_1_ toxicity in human blood. Cytotechnology.

[99] Johansen MB, Kiemer L, Brunak S (2006). Analysis and prediction of mammalian protein glycation. Glycobiology.

[100] Price AK, de Godoy MRC, Harper TA, Knap KE, Joslyn S, Pietrzkowski Z, Cross BK, Detweiler KB, Swanson KS (2017). Effects of dietary calcium fructoborate supplementation on joint comfort and flexibility and serum inflammatory markers in dogs with osteoarthritis. J Anim Sci.

[101] Geroldi D, Falcone C, Emanuele E (2006). Soluble receptor for advanced glycation end products: from disease marker to potential therapeutic target. Curr Med Chem.

[102] Luevano-Contreras C, Chapman-Novakofski K (2010). Dietary advanced glycation end products and aging. Nutrients.

[103] Kellow NJ, Savige GS (2013). Dietary advanced glycation end-product restriction for the attenuation of insulin resistance, oxidative stress and endothelial dysfunction: a systematic review. Eur J Clin Nutr.

[104] Kaneshima H, Kitsutaka T, Akagi M (1968). Studies on metabolic effects of borate (VII): effects of borate on the reduction of methemoglobin (2). Food Hyg Saf Sci (Shokuhin Eiseigaku Zasshi).

[105] Çelikezen FÇ, Turkez H, Togar B, Izgi MS (2014). DNA damaging and biochemical effects of potassium tetraborate. EXCLI J.

[106] Massie HR (1994). Effect of dietary boron on the aging process. Environ Health Perspect.

[107] Biego GH, Joyeux M, Hartemann P, Debry G (1998). Daily intake of essential minerals and metallic micropollutants from foods in France. Sci Total Environ.

[108] Yazbeck C, Kloppmann W, Cottier R, Sahuquillo J, Debotte G, Huel G (2005). Health impact evaluation of boron in drinking water: a geographical risk assessment in Northern France. Environ Geochem Health.

[109] Zhang W, Ogando DG, Bonanno JA, Obukhov AG (2015). Human SLC4A11 is a novel NH_3_/H^+^ co-transporter. J Biol Chem.

[110] Park M, Li Q, Shcheynikov N, Muallem S, Zeng W (2005). Borate transport and cell growth and proliferation. Not only in plants. Cell Cycle.

[111] Kaya A, Karakaya HC, Fomenko DE, Gladyshev VN, Koc A (2009). Identification of a novel system for boron transport: Atr1 is a main boron exporter in yeast. Mol Cell Biol.

[112] Weerasinghe AJ, Amin SA, Barker RA, Othman T, Romano AN, Parker Siburt CJ, Tisnado J, Lambert LA, Huxford T, Carrano CJ, Crumbliss AL (2013). Borate as a synergistic anion for *Marinobacter algicola* ferric binding protein, FbpA: a role for boron in iron transport in marine life. J Am Chem Soc.

[113] Dembitsky VM, Al Quntar AA, Srebnik M (2011). Natural and synthetic small boron-containing molecules as potential inhibitors of bacterial and fungal quorum sensing. Chem Rev.

